# Cerebrospinal fluid proteomic study of two bipolar disorder cohorts

**DOI:** 10.1038/s41380-022-01724-2

**Published:** 2022-08-19

**Authors:** Anniella Isgren, Andreas Göteson, Jessica Holmén-Larsson, Aurimantas Pelanis, Carl Sellgren, Erik Joas, Timea Sparding, Henrik Zetterberg, Erik Smedler, Joel Jakobsson, Mikael Landén

**Affiliations:** 1 Institute of Neuroscience and Physiology, Department of Psychiatry and Neurochemistry, Sahlgrenska Academy at University of Gothenburg, Gothenburg and Mölndal, Gothenburg, Sweden; 2 Department of Physiology & Pharmacology, Karolinska Institutet, Stockholm, Sweden; 3 Centre for Psychiatry Research, Department of Clinical Neuroscience, Karolinska Institutet, Stockholm & Stockholm Health Care Services, Region Stockholm, Stockholm, Sweden; 4 Department of Neurodegenerative Disease, UCL Institute of Neurology, London, UK; 5 Clinical Neurochemistry Laboratory, Sahlgrenska University Hospital, Mölndal, Gothenburg, Sweden; 6 UK Dementia Research Institute at UCL, London, UK; 7 Hong Kong Center for Neurodegenerative Diseases, Hong Kong, China; 8 Department of Medical Epidemiology and Biostatistics, Karolinska Institutet, Stockholm, Sweden

**Keywords:** Bipolar disorder, Neuroscience

## Abstract

The pathophysiology of bipolar disorder remains to be elucidated and there are no diagnostic or prognostic biomarkers for the condition. In this explorative proteomic study, we analyzed 201 proteins in cerebrospinal fluid (CSF) from mood stable bipolar disorder patients and control subjects sampled from two independent cohorts, amounting to a total of 204 patients and 144 controls. We used three Olink Multiplex panels, whereof one specifically targets immune biomarkers, to assess a broad set of CSF protein concentrations. After quality control and removal of proteins with a low detection rate, 105 proteins remained for analyses in relation to case–control status and clinical variables. Only case–control differences that replicated across cohorts were considered. Results adjusted for potential confounders showed that CSF concentrations of growth hormone were lower in bipolar disorder compared with controls in both cohorts. The effect size was larger when the analysis was restricted to bipolar disorder type 1 and controls. We found no indications of immune activation or other aberrations. Growth hormone exerts many effects in the central nervous system and our findings suggest that growth hormone might be implicated in the pathophysiology of bipolar disorder.

## Introduction

Bipolar disorder is a lifelong condition characterized by recurrent fluctuations in mood state and energy, affecting more than 1% of the world’s population [1]. Even though our understanding of the pathophysiology has improved over the past decades, the biological basis of the disease remains largely unknown [1, 2]. Moreover, there are currently no valid biomarkers to aid the diagnostic process that currently relies on clinical assessments.

One means to investigate brain pathophysiology is the study of proteins in cerebrospinal fluid (CSF). Protein concentrations in CSF might reflect central nervous system (CNS) processes more directly than serum or plasma due to the relative impermeability of the blood–CSF and blood-brain barriers [3–5]. CSF is, however, cumbersome to collect and such studies are rarer than serum studies. A meta-analysis identified 34 CSF studies of bipolar disorder, investigating a total of 117 unique biomarkers. Only two findings (elevated homovanillic acid and 5-hydroxy-indoleacetic acid) replicated across studies [6]. To unearth novel findings, hypothesis-driven research needs to be complemented with studies surveying large sets of biomarkers.

We recently analyzed CSF samples from the same two independent bipolar cohorts used in the present study with a multiplex protein assay targeted for CNS processes [7]. Two proteins (testican-1 and C-type lectin domain family 1 member B) differed between patients and controls. In the present study, we survey a broader set of CSF proteins by using three Olink Multiplex panels, whereof one specifically targets immune markers. Although pro-inflammatory immune dysregulation and microglia activation have been implicated in bipolar disorder [8–11], findings are conflicting and the role of immune dysfunction in bipolar disorder is as yet undecided [6, 9, 11]. Our primary aim was to test if CSF protein concentrations differ between patients with bipolar disorder and controls in two independent cohorts. The secondary aim was to investigate if case-control associated CSF protein concentrations associate with clinical subphenotypes.

## Patients and methods

### Study population

The study population is derived from the St. Göran bipolar project (SBP), which comprises two independently collected cohorts of patients with bipolar disorder and controls. The first case–control cohort was collected in Stockholm, Sweden (SBP-S) and the second in Gothenburg, Sweden (SBP-G). Patients in the SBP-S cohort (*n* = 134) were recruited from the Northern Stockholm Psychiatric Clinic, Stockholm, Sweden. The SBP-G cohort (*n* = 70) was recruited from the Bipolar Clinic at the Sahlgrenska University Hospital, Gothenburg, Sweden. Inclusion criteria were patients at least 18 years old meeting the DSM-IV-TR criteria for any bipolar spectrum disorder.

The recruitment process and work-up procedures for patients and selection of control subjects have been described in detail previously [7, 12–14]. In brief, an adapted Swedish version of the semi-structured interview Affective Disorder Evaluation (ADE) was used to aid the diagnosis of bipolar disorder. The ADE was developed for the Systematic Treatment Enhancement Program of Bipolar Disorder (STEP-BD) project [15] and includes adapted versions of the mood modules of the Structured Clinical Interview for DSM-IV. Co-morbid psychiatric disorders were screened for using the Mini International Neuropsychiatric Interview (M.I.N.I.) [16]. The ADE and M.I.N.I. interviews were conducted by psychiatrists or residents in psychiatry at the Stockholm center, and by psychiatrists, residents in psychiatry, a psychologist, and a research nurse at the Gothenburg center. Board-certified psychiatrists specialized in bipolar disorder made the final best-estimate diagnostic decision [17, 18] based on previous medical records, ADE, M.I.N.I., and interviews with next of kin where possible. Disease severity was assessed using the Clinical Global Impression (CGI) rating scale, and the Global Assessment of Functioning (GAF) scale divided into functional level (GAF-f) and symptom severity (GAF-s).

The control participants (*n* = 89 in the SBP-S; *n* = 55 in the SBP-G) were randomly selected by Statistics Sweden from the population residing in the same respective catchment areas where patients were recruited. Controls were first screened over phone for inclusion and exclusion criteria by a research nurse. At the subsequent scheduled visit, a psychiatrist or resident in psychiatry conducted an interview using M.I.N.I. to exclude psychiatric disorders. The Alcohol Use Disorders Identification Test (AUDIT) and Drug Use Disorders Identification Test (DUDIT) questionnaires were used to screen for alcohol or drug abuse. Controls underwent somatic examination, lumbar puncture, and blood tests under the same protocol as patients. Exclusion criteria for controls were schizophrenia or bipolar disorder in first-degree relatives, untreated endocrinological disorders, pregnancy, overconsumption of alcohol (defined as elevated concentrations of carbohydrate deficient transferrin or >8 standard drinks per time more than two times per week), any other substance abuse, dementia, neurological conditions other than mild migraines, and any psychiatric condition other than past minor depressive episodes, past isolated episodes of panic disorder, past mild eating or obsessive–compulsive disorder that remitted spontaneously or with brief psychotherapy counseling.

The study was approved by the Regional Ethics Committee in Stockholm and carried out in accordance with the Declaration of Helsinki. All participants gave written and oral consent to participate in the study. Patients were in a euthymic state when the consent was obtained.

### Cerebrospinal fluid collection

Cerebrospinal fluid was collected by lumbar puncture between 09.00 and 10.00 am following an overnight fast. Patients were in a stable mood (i.e., not suffering from an acute depressive, manic, hypomanic, or mixed episode) as judged by the treating physician. Subsyndromal mood symptoms were assessed by the Montgomery-Åsberg Depression Rating Scale (MADRS) and the Young Mania Rating Scale (YMRS). A total of 12 mL CSF was collected, inverted to avoid gradient effects, divided into aliquots, and stored at −80 °C pending analyses. CSF collection from control subjects followed the same procedure. In the SBP-S, no samples were centrifugated except six control samples. In the SBP-G, all samples were centrifugated. All samples in the study were thawed and refrozen once before analysis. In the SBP-S, CSF sampling occurred between 2005 and 2008 for patients and between 2009 and 2011 for controls. In the SBP-G, CSF was collected between 2010 and 2015 for patients and between 2012 and 2014 for controls. Patients remained on their prescribed medication.

### Immunoassays and preprocessing of protein data

Protein concentrations in CSF were measured using multiplex immunoassays based on proximity extension assay (PEA) technology (Olink^®^ Proteomics, Uppsala, Sweden), which has been described in detail elsewhere [19]. Briefly, the PEA method includes oligonucleotide-labeled antibodies that bind to the target protein. This process is coupled with a qPCR readout, which enables a multiplex setup with low cross-reactivity [19]. For this study, we assessed a broad set of proteins using three Olink Multiplex panels: Inflammation I, Oncology I, and Cardiovascular I. Combined, these panels include 201 unique protein assays. Six assays overlapped with a previous study conducted in the same study population [7]. Correlations for the overlapping assays are presented in Supplementary figure 1 and case-control comparisons for these proteins are shown in supplementary table 1. Analyses, initial preprocessing, and quality control were performed at the Clinical Biomarker Facility at SciLifeLab Uppsala, Sweden (SBP-S) and at Olink Bioscience Uppsala, Sweden (SBP-G). Samples from the SBP-S were analyzed on three plates and samples from the SBP-G on two plates. Samples from patients and controls were evenly distributed across the plates. Protein concentrations were represented by NPX (normalized protein expression) values on an arbitrary log2-scale [19]. Brain-derived neurotrophic factor (BDNF) was excluded in both cohorts due to technical issues. No samples failed the initial quality control in either cohort. The staff performing the analyses were blinded to all phenotype information. After data delivery, we performed additional preprocessing and quality control steps. The NPX values were median-centered per protein and plate to adjust for batch effect, and subsequently autoscaled using the standard deviation as the scaling factor [20]. Sample quality was checked using principal component analyses and by comparing protein concentrations for panel-overlapping assays. No samples failed this quality control.

Among the 65 proteins that were included in two or three panels, the panel with most values over the limit of detection (LOD) was selected [19]. The protein assays that overlapped across panels correlated strongly (median rho = 0.87 in both the SBP-S and SBP-G). The proteins with >25% values below LOD in both the patient and the control groups (*n* = 96 in both the SBP-S and SBP-G) were excluded from further analysis in each cohort. For the remaining proteins, values below the LOD were kept. The associations between time for CSF sampling and protein concentrations were tested by Spearman correlation. No protein concentration was significantly correlated with time for CSF sampling in both cohorts. Four of the samples in the SBP-S were suspected to have been contaminated with blood at lumbar puncture due to either high red blood cell count (>500 per microliter) or a protein profile similar to blood. These samples were excluded in a sensitivity analysis. The protein dataset used for final analyses contained 105 proteins in each cohort (98 of these proteins were overlapping between cohorts). All proteins included in the study are listed in Supplementary table 2.

### Analysis of albumin concentrations

Serum and CSF concentrations of albumin were measured by immunonephelometry on a Beckman IMMAGE Immunochemistry system (Beckman Instruments, Beckman Coulter, Brea, CA, USA) at the Clinical Neurochemistry Laboratory in Mölndal, Sweden, using a method accredited by the Swedish Board for Accreditation and Conformity Assessment (SWEDAC). Intra- and inter-assay coefficients of variation were below 10%. The ratio between the albumin concentration in CSF (mg/L) and serum (g/L) was calculated and used to assess blood–brain barrier function [21].

### Statistical analyses

We processed and analyzed data in R (version 3.6.3). Code is available from the corresponding author upon request. Statistical analyses were conducted separately in each cohort. The level of significance was set at *p* < 0.05. Demographic and clinical group differences were established by Pearson’s Chi-squared test or Kruskal-Wallis rank sum test. Principal component analysis was used to summarize the variance of all included proteins. Spearman’s rank-order correlation were used to test associations between the first principal component and demographic factors, and to calculate correlations between CSF and serum protein concentrations (measured in a previous study by us [22]).

We built covariate-adjusted logistic regression models to test case–control differences in concentrations of the measured proteins. To decrease the risk for type I errors, we only considered statistically significant findings that replicated across the two independent cohorts instead of adjusting p-values for multiple testing. Potential confounders that were associated with case–control status in one or both cohorts (age, sex, body mass index [BMI], CSF/serum albumin ratio, and nicotine use) were included as covariates in the regression models. The proportion of diagnostic subgroups (e.g., bipolar disorder type 1 and type 2) differed between the two cohorts. We, therefore, conducted a secondary analysis compromising only patients with bipolar disorder type 1 (i.e., the prototypical bipolar disorder subtype) and controls. Also, associations between all protein concentrations and psychiatric drugs were tested using covariate-adjusted linear regression with protein concentration as the dependent variable.

In *post hoc* analyses of growth hormone (GH), we tested associations between GH and the clinical features past depressive episodes, past (hypo-)manic episodes, CGI, GAF-f, and GAF-s using covariate-adjusted ordinal regressions with the clinical feature as dependent variable. Clinical variables were categorized due to skewed distribution and/or outliers.

## Results

### Demographics and clinical characteristics

Demographics of the study populations and clinical characteristics of the patients are presented in Table 1. The sex distribution did not differ between patients and controls but female sex was more common at both study sites. More patients than controls used nicotine (both sites), and patients had higher BMI than controls in the SBP-S. Patients in the SBP-S reported fewer lifetime mood episodes, had more previous psychotic episodes, scored lower on CGI at interview, had less comorbid anxiety disorders, and used less anticonvulsants than patients in the SBP-G.

**Table 1:** Demographics and clinical characteristics of the study population.

	SBP-S			SBP-G		
	Patients (*N* = 134)	Controls (*N* = 89)	*p*	Patients (*N* = 70)	Controls (*N* = 55)	*p*
**Demographics**						
Male sex	53 (40%)	41 (46%)	0.335^a^	25 (36%)	25 (45%)	0.270^a^
Age, years	35.5 (29.0, 50.8)	36.0 (28.0, 47.0)	0.500^b^	38.5 (29.2, 49.0)	45.0 (32.0, 52.0)	0.077^b^
BMI	24.8 (22.2, 27.7)^d^	23.4 (21.6, 25.5)	**0.006**^b^	25.0 (23.0, 28.4)	25.0 (22.2, 26.7)	0.178^b^
Nicotine use	57 (47%)^e^	21 (24%)	**<0.001**^a^	30 (45%)^f^	14 (25%)	**0.027**^a^
CSF/serum albumin ratio	5.2 (4.2, 6.6)	4.8 (3.7, 6.0)^d^	**0.015**^b^	4.7 (3.7, 6.5)	4.6 (3.5, 5.5)	0.474^b^
**Patient clinical characteristics**						
BD subtypes:						0.119^a^
BD 1	65 (49%)			27 (39%)		
BD 2	51 (38%)			37 (53%)		
BD spectrum^c^	18 (13%)			6 (9%)		
Duration of illness, years	16 (9, 27)^h^			18 (11, 30)^f^		0.204^b^
Total lifetime mood episodes:	11 (6, 23)^g^			25 (12, 38)^f^		**<0.001**^b^
Depressive episodes	4 (3, 10)^g^			10 (5, 15)^f^		**<0.001**^b^
(Hypo)manic episodes	4 (2, 10)^h^			10 (4, 20)^f^		**<0.001**^b^
Mixed episodes	0 (0, 0)^h^			0 (0, 10)^f^		**<0.001**^b^
Ever psychotic	67 (52%)			17 (25%)		**<0.001**^a^
Any suicide attempt or self-harm	54 (42%)			25 (37%)		0.510^a^
CGI lifetime	4 (4, 5)^i^			4 (4, 5)^j^		0.380^b^
CGI at interview	2 (2, 4)^g^			4 (2, 5)^i^		**<0.001**^b^
GAF-s	68 (60, 71)^h^			60 (55, 65)^n^		**<0.001**^b^
GAF-f	68 (70, 71)^h^			59 (55, 65)^n^		**<0.001**^b^
MADRS at sampling	4 (1, 11)^k^			3 (1, 9)		0.417^b^
YMRS at sampling	0 (0, 2)^l^			1 (0, 2)		0.065^b^
Comorbidities:						
Anxiety disorder	20 (15%)^i^			26 (37%)^f^		**<0.001**^a^
Personality disorder	3 (2%)^g^			1 (1%)^f^		0.677^a^
ADHD	9 (7%)^m^			2 (3%)^f^		0.203^a^
Alcohol abuse	16 (12%)^j^			3 (4%)^f^		0.068^a^
Substance abuse	6 (5%)^j^			1 (1%)^f^		0.215^a^
Medications:						
Lithium	79 (59%)			35 (50%)		0.221^a^
Antipsychotics	33 (25%)			23 (33%)		0.211^a^
Anticonvulsants	47 (35%)			35 (50%)		**0.039**^a^
Antidepressants	63 (47%)			35 (50%)		0.685^a^

Data shown is median value with interquartile range in brackets, or number of individuals with percentage in brackets.

*p*-values smaller than 0.05 are shown in bold.

*ADHD* attention-deficit/hyperactivity disorder, *BD* bipolar disorder, *BMI* body mass index, *CGI* Clinical Global Impression rating scale, *GAF* Global Assessment of Functioning scale divided into functional level (GAF-f) and symptom severity (GAF-s), *MADRS* Montgomery-Åsberg Depression Rating Scale, *SBP-G* St. Göran bipolar project Gothenburg, *SBP-S* St. Göran bipolar project Stockholm, *YMRS* Young Mania Rating Scale.

^a^Pearson’s Chi-squared test. ^b^Kruskal–Wallis rank sum test.

^c^BD spectrum groups consisted of 2/2 patients with cyclothymia, 6/1patients with mania/hypomania due to antidepressant drug treatment, 0/1 patients with recurrent depression in persons with hyperthymic temperament, 1/1 patients with recurrent depressions that are admixed with dysphoric hypomania, 2/0 patients with schizoaffective disorder bipolar type, and 7/1 patients with bipolar disorder not otherwise specified in SBP-S/SBP-G.

^d^Data missing for one individual. ^e^Data missing for 12 individuals. ^f^Data missing for three individuals. ^g^Data missing for seven individuals. ^h^Data missing for five individuals. ^i^Data missing for six individuals. ^j^Data missing for eight individuals. ^k^Data missing for 22 individuals. ^l^Data missing for 26 individuals. ^m^Data missing for ten individuals. ^n^Data missing for four individuals.

### Description of the protein dataset

There was a positive correlation between the vast majority of proteins (rho median [interquartile range] = 0.47 [0.32–0.60] in the SBP-S and 0.48 [0.33–0.64] in the SBP-G). In principal component analyses (Supplementary figure 2), the first principal component explained 49% and 53% of the variation in the data and the second principal component explained 7% and 6% for the SBP-S and SBP-G, respectively. The first principal component correlated with age (both cohorts), CSF/serum albumin ratio (both cohorts), sex (both cohorts), BMI (SBP-S), but not with nicotine use (Supplementary figure 2).

### Patient-control comparisons

In a principal component analysis, there was no group separation between patients and controls when considering all 105 proteins together (Supplementary figure 3). When analyzing the 105 proteins separately, the concentrations of fourteen CSF proteins in the SBP-S and nine proteins in the SBP-G differed significantly between patients and controls (p-value<0.05, Supplementary table 3). For eight of these proteins, there was a significant difference in one cohort, and an estimate in the same direction (however not statistically significant) in the other cohort. Of these proteins, eotaxin-1 (CCL11), placenta growth factor (PGF), C-X-C motif chemokine 1 (CXCL1), C-X-C motif chemokine 6 (CXCL6), and prolactin had higher concentrations in patients compared with controls and proteinase-activated receptor 1 (PAR-1), epidermal growth factor receptor (EGFR), and prostasin (PRSS8) had lower concentrations in patients compared with controls. A graph of protein–protein interactions for these proteins (and GH) from the STRING database of known and predicted protein–protein interactions [23] is shown in Supplementary figure 4.

**Fig. 1:**
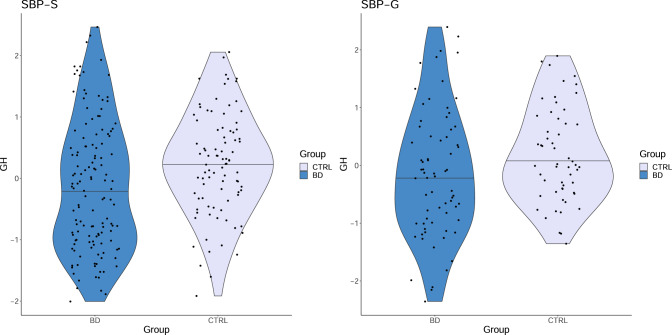
Violin plots showing cerebrospinal fluid concentrations of growth hormone (GH) in patients with bipolar disorder and in controls in both cohorts. Normalized protein expression values on y-axis. BD bipolar disorder, CTRL control, SBP-G St. Göran bipolar project Gothenburg, SBP-S St. Göran bipolar project Stockholm.

The only protein that replicated across both cohorts was GH, which was lower in bipolar disorder patients than controls (see Table 2 for the adjusted logistic regression models). Figure 1 shows the distribution of GH concentrations in patients and controls. In a secondary analysis, comparing only patients with bipolar disorder type 1 and controls, GH was again the only protein with a significant association between CSF concentrations and bipolar disorder in both cohorts, and with larger effect sizes than in the analysis including all patients (SBP-S: β = −0.56, *p* = 0.046; SBP-G: β = −1.75, *p* < 0.001, see Supplementary table 4).

**Table 2:** Logistic regressions showing the association between growth hormone (GH) and bipolar disorder.

SBP-S (*n* = 209)	β	SE	OR	95% CI	*p*
GH	−0.47	0.22	0.63	0.40–0.96	**0.035**
Age	−0.01	0.01	0.99	0.97–1.01	0.455
Male sex	−0.89	0.35	0.41	0.20–0.80	**0.011**
BMI	0.039	0.051	1.04	0.94–1.15	0.443
Nicotine use	0.99	0.32	2.69	1.44–5.16	**0.002**
Albumin ratio	0.17	0.078	1.18	1.02–1.38	**0.034**
**SBP-G** (***n* = 122)**	**β**	**SE**	**OR**	**95% CI**	***p***
GH	−0.69	0.32	0.50	0.26–0.93	**0.033**
Age	−0.05	0.02	0.95	0.91–0.98	**0.006**
Male sex	−1.20	0.49	0.30	0.11–0.77	**0.015**
BMI	0.013	0.065	1.01	0.89–1.15	0.840
Nicotine use	0.96	0.44	2.60	1.13–6.30	**0.028**
Albumin ratio	0.10	0.12	1.10	0.87-1.41	0.424

Case–control status is dependent variable. Age, sex, BMI, CSF/serum albumin ratio and nicotine use are covariates.

*p*-values smaller than 0.05 are shown in bold.

*BMI* body mass index, *CI* confidence interval, *CSF* cerebrospinal fluid, *OR* odds ratio, *SE* standard error, *SBP-G* St. Göran bipolar project Gothenburg, S*BP-S* St. Göran bipolar project Stockholm.

### Post hoc analyses of GH

We conducted *post hoc* analyses given that CSF concentrations of GH were significantly lower in patients with bipolar disorder compared with controls in both cohorts. GH was negatively correlated with age (SBP-S: rho = −0.43, *p* < 0.001; SBP-G: rho = −0.35, *p* < 0.001), BMI (SBP-S: rho = −0.63, *p* < 0.001; SBP-G: rho = −0.68, *p* < 0.001), and CSF/serum albumin ratio (SBP-S: rho = −0.31, *p* < 0.001; SBP-G: rho = −0.34, *p* < 0.001), and positively associated with female sex (SBP-S: rho=0.40, *p* < 0.001; SBP-G: rho=0.47, *p* < 0.001). GH was not associated with nicotine use in either cohort.

In relation to bipolar disorder features, CSF GH concentration associated negatively with previous depressive episodes in the SBP-S (OR = 0.59, *p* = 0.026), but not in the SBP-G (OR = 1.21, *p* = 0.620). There was no association with previous (hypo)manic episodes, psychotic symptoms, CGI, GAF-s, or GAF-f. Antipsychotic treatment associated negatively with CSF GH concentrations in the SBP-G (β = −0.84, *p* < 0.001), but not in the SBP-S (β = −0.20, *p* = 0.30). Treatment with lithium, anticonvulsants, or antidepressants was not associated with CSF GH concentrations. The regression models with CSF GH and clinical features and drugs are shown in Supplementary table 5. In a sensitivity analysis where we excluded patients with antipsychotic treatment, the significant negative association between CSF GH concentrations and bipolar disorder remained in the SBP-S (β = −0.57, *p* = 0.019), but was no longer significant in the SBP-G (β = −0.43, *p* = 0.209) (Supplementary table 6).

In a second sensitivity analysis, we removed the six centrifugated control samples and four samples with suspected blood contamination in the SBP-S. The main finding of an association between lower CSF GH and bipolar disorder remained significant (OR = 0.63; *p* = 0.023). Finally, we performed a third sensitivity analysis, where we excluded patients with a MADRS score of 13 or higher (26 patients in the SBP-S and nine patients in the SBP-G), and/or YMRS score of 14 or higher (one patient in the SBP-G). Also in this analysis, we found an association between lower CSF GH and bipolar disorder in both cohorts, with larger effect sizes than in the main analyses (Supplementary table 7).

### Associations between CSF protein concentrations and psychiatric drugs

Seven proteins were significantly associated with one of the psychiatric drug groups in both the SBP-S and SBP-G. Positive drug associations were seen for prolactin (antipsychotics), placenta growth factor (PGF) (lithium), monocyte chemotactic protein 1 (MCP-1) (lithium), interleukin-8 (IL-8) (lithium), folate receptor alpha (FR-alpha) (lithium), and cathepsin D (CTSD) (lithium). Lithium was negatively associated with fibroblast growth factor 19 (FGF-19) in both cohorts. Results from linear regression models are shown in Supplementary table 8 and a heatmap visualizing the associations between CSF protein concentrations and psychiatric drugs are shown in Supplementary figure 5.

### Associations between CSF and serum protein concentrations

Correlation analyses showed that for each protein separately, the correlations between the CSF concentrations in this study, and the serum concentrations measured in a previous study by us (CSF and blood sampling were performed at the same day) [22], were generally low (median rho 0.18 in the SBP-S and 0.12 in the SBP-G). For GH, the between–fluid correlation coefficient was 0.59 in the SBP-S and 0.55 in the SBP-G. Correlations between CSF and serum concentrations are shown in Supplementary table 9 and in Supplementary figure 6.

## Discussion

We conducted an exploratory proteomic study of bipolar disorder using CSF collected in two independent case–control cohorts. This is the second study using the PEA technology to measure CSF protein concentrations in these cohorts [7]. Here, we used three Olink protein biomarker discovery panels, whereof one was targeted toward immune markers. Out of a total of 201 proteins, whereof 105 remained after quality control and excluding proteins with low detection rate, the only finding that replicated across cohorts was an association between lower CSF concentrations of GH and bipolar disorder. This association was stronger when restricting the analysis to bipolar disorder type 1 (the prototypical type of bipolar disorder) and controls.

Growth hormone is produced by somatotropic cells of the anterior pituitary gland [24] and exerts many effects in the CNS, not only during the fetal and childhood periods but also in adulthood [25]. Receptors for GH are expressed in the human brain, including in the choroid plexus, hypothalamus, hippocampus, the pituitary, putamen, and thalamus [26]. Not only has GH been implicated in promoting glial differentiation, myelination, brain growth, and neuronal plasticity [25], but GH has also been shown to influence higher mental functions such as memory, behavior, mood, working ability, and alertness [25, 27]. There is further an interesting theoretical connection between GH and the circadian rhythm dysfunction associated with bipolar disorder [28, 29] through the suggested crosstalk between the GH/insulin-like growth factor 1 (IGF-1) axis and the circadian clock system [30]. Interestingly, several studies have found increased peripheral IGF-1—a negative feedback regulator of GH gene expression [31]—in patients with bipolar disorder [32, 33].

Although no previous study has assessed CSF concentrations of GH in bipolar disorder, there are studies of peripheral GH in psychiatric disorders. One study found higher IGF-1 and slightly lower GH (not statistically significant) in serum from bipolar disorder patients compared with controls [34]. Further, one study found lower clonidine-induced GH stimulation in bipolar and schizoaffective disorder, but not in schizophrenia, compared with controls [35]. In depressed patients, studies have shown a blunted GH response to physical stimuli [36], as well as an altered circadian GH secretion pattern [27]. In the present study, previous number of depressive episodes was the only clinical feature that associated with CSF GH concentrations in one (SBP-S) but not the other (SBP-G) cohort.

Patients in this study remained on their prescribed medications, which might have influenced CSF protein concentrations. CSF GH concentrations correlated negatively with antipsychotic use in the SBP-G but not in the SBP-S cohort. When excluding patients with antipsychotic treatment, the negative association between CSF GH concentrations and bipolar disorder remained in the SBP-S but fell below the statistical threshold in the SBP-G (a smaller sample size contributing to this). Previous studies on the influence of antipsychotics on GH concentrations are scarce and restricted to serum. While one study found a reduced GH peak during sleep for patients on olanzapine [37], another study found no associations between GH and olanzapine treatment [38], and a third study found increased GH concentrations during antipsychotic treatment [39]. Taken together, our findings suggest that antipsychotic drug use cannot alone explain the association between CSF GH and bipolar disorder.

One of our protein panels targeted immune markers and many of the studied proteins have immunological roles. Notably, no immune marker differences between patients and controls replicated across cohorts. We have previously shown higher CSF concentrations of the immune-related proteins IL-8, MCP-1, and chitinase-3-like protein 1 (YKL-40/CHI3L1) in bipolar disorder compared with controls in the SBP-S cohort using other analytical methods (see Supplementary table 10 for a comparison between the methods and results of the present study and the previously published studies) [5, 40]. These previous findings did not replicate in the SBP-G cohort, which could be due to previous false positives, case-mix differences, or unmeasured confounding factors influencing the CSF proteome. However, IL-8 and MCP-1 (and three other proteins) concentrations were relatively higher in patients with ongoing lithium treatment in both cohorts indicating a possible connection between lithium treatment and the immune system.

It is noteworthy that this proteomic CSF study of bipolar disorder did not reveal any specific aberrations apart from the GH finding. Bipolar disorder hence differs from neurodegenerative CNS disorders such as multiple sclerosis and Alzheimer’s disease, where evident CSF aberrations have been found using the same PEA proteomic technology [41, 42]. Bipolar disorders possibly encompass a pathophysiologically heterogeneous group of diseases, but our subgroup analysis comprising only bipolar I disorder patients and controls was equally negative except from the GH finding. Although we surveyed a large number of proteins, our assays by no means cover the whole CSF proteome. Other methods (e.g., exploratory mass spectrometry) that capture even greater number of proteins might still reveal CSF biomarkers for bipolar disorder. Finally, we sampled CSF when patients were in a stable mood. It is possible that more pronounced changes in the CSF proteome occur during acute mania or depression. A challenging but potentially informative future route would thus be to sample CSF across different mood states.

A main strength of this study is that protein concentrations were measured in CSF instead of in blood which might increase CNS specificity. We showed that the between–fluid correlation for CSF and serum protein concentrations were low for the vast majority of proteins and CSF might reflect brain processes more closely compared with serum. The meticulous clinical examinations ensure diagnostic accuracy and allowed us to control for several potential confounders. Further, we sampled two independent case–control cohorts, which allowed us to replicate—or to not replicate—findings. The issue of low replication rates within the field of CSF studies of bipolar disorder was demonstrated in the previously mentioned review by Knorr et al. Indeed, when we analyzed the two cohorts separately, we found that fourteen and nine proteins differed significantly between patients and controls—but only GH replicated across the two cohorts. Even though the case–control differences seen in only one of the cohorts are not false positives by definition, they are likely to be of less significance. Hence, our use of a replication cohort improved the robustness of results by reducing the risk for false positives.

A general limitation of CSF and other biofluid studies is that factors unrelated to bipolar disorder may affect protein concentrations. Even though we adjusted for several potential confounders, there might be residual unmeasured confounding factors. Second, the cross-sectional design hampers causal conclusions. Third, CSF sampling from patients preceded the collection from controls in the SBP-S cohort and long-term storage can affect protein concentrations [43]. However, no protein showed consistent correlation with CSF sampling date in our study. Fourth, the pre-analytical handling of samples differed between the two cohorts with respect to centrifugation. We therefore conducted a sensitivity analysis in the SBP-S with non-centrifugated samples only and the main finding remained. Finally, we could not control for the use of psychotropic drugs in comparisons between patients and controls. However, CSF GH concentrations were not consistently associated with major psychotropic drug groups.

In conclusion, this exploratory CSF study of two bipolar disorder cohorts demonstrates lower CSF GH concentrations in patients with bipolar disorder compared with controls. Growth hormone exerts many effects in the brain and our findings suggest that GH might be implicated in the pathophysiology of bipolar disorder.

## Supplementary information

Supplemental material 1

Supplemental material 2
