# Supplementary material for: Cerebrospinal fluid proteomic study of two bipolar disorder cohorts
Source: Mol Psychiatry. 2022 Aug 19;27(11):4568–74. doi: 10.1038/s41380-022-01724-2 (PMC9734044; doi:10.1038/s41380-022-01724-2)
Supplement: Supplementary file 1 — Supplemental material 1 [file 41380_2022_1724_MOESM1_ESM.docx]

**Supplementary Information - Cerebrospinal fluid proteomic study of two bipolar disorder cohorts**

*Suppl*_*material_1_revised* (this file) contains:

| Index |  |
| --- | --- |
| p. 4 | **Supplementary table 1**. Case-control comparisons for the six assays included both in the present study and in a previously published study (Göteson et al.). |
| p. 6 | **Supplementary table 5**. Linear and ordinal regressions for cerebrospinal fluid growth hormone and clinical variables. |
| p. 7 | **Supplementary table 6**. Logistic regressions showing the association between cerebrospinal fluid growth hormone and bipolar disorder, when patients with antipsychotic treatment were excluded. |
| p. 8 | **Supplementary table 7**. Logistic regressions showing the association between growth hormone and bipolar disorder, when patients with MADRS score of 13 or higher, and/or YMRS score of 14 or higher were excluded. |
| p. 9 | **Supplementary table 10**. Summary of methods and results of the present study and results from two previous studies measuring CSF concentrations of IL-8, MCP-1, and YKL-40 in bipolar disorder patients and controls. |
| p. 11 | **Supplementary figure 1**. Correlations between protein concentrations in the present study and in a previously published study (Göteson et al.) for the six overlapping assays. |
| p. 12 | **Supplementary figure 2**. Principal components and their relations to demographic variables. |
| p. 13 | **Supplementary figure 3**. Principal component analyses of cerebrospinal fluid protein concentrations in bipolar disorder patients and controls. |
| p. 14 | **Supplementary figure 4**. Graph of protein–protein interactions for GH and the eight proteins for which there was a significant difference in CSF concentrations between patients and controls in one cohort, and an estimate in the same direction (however not statistically significant) in the other cohort. Results from STRING database of known and predicted protein–protein interactions. |
| p. 15 | **Supplementary figure 5**. Heatmap visualizing the associations between cerebrospinal fluid protein concentrations and psychiatric drug groups. |
| p. 16 | **Supplementary figure 6**. Histogram of between–fluid (CSF–serum) protein correlations. |

*Suppl_material_2_revised.xlsx* contains:

| Index |  |
| --- | --- |
| Sheet 1 | **Supplementary table 2 (S2)**. List of proteins included in the study. All proteins that are measured are included in the list. “Yes” and “No” indicates if the protein is included in data analysis or not (if not included in data analysis the reason is that a large proportion of individuals had values below the limit of detection). |
| Sheet 2 | **Supplementary table 3 (S3)**. Case–control comparisons. Results from all logistic regressions investigation the associations between cerebrospinal fluid protein concentrations and case-control. Case-control is the dependent variable. Covariates: Age, sex, body mass index, CSF/serum albumin ratio, nicotine use. |
| Sheet 3 | **Supplementary table 4 (S4)**. Bipolar type 1 – control comparisons. Results from all logistic regressions investigation the associations between cerebrospinal fluid protein concentrations and case–control, when the patient groups were restricted to bipolar disorder type 1. Case–control is the dependent variable. Covariates: Age, sex, body mass index, CSF/serum albumin ratio, nicotine use. |
| Sheet 4 | **Supplementary table 8 (S8)**. Associations between cerebrospinal fluid proteins and drug groups. Results from linear regression models with protein concentrations as the dependent variable. Covariates: Age, sex, CSF/albumin ratio. *Abbreviations*: AC, anticonvulsants. AP, antipsychotics. AD, antidepressants. Li, lithium. |
| Sheet 5 | **Supplementary table 9 (S9)**. Spearman correlations between protein concentrations in CSF and serum. Correlation coefficients (rho) and p-values are shown. |

**Supplementary table 1**. Case-control comparisons for the six assays included both in the present study and in a previously published study (Göteson et al.^1^).

|  | **Isgren et al. (present study)** | | | **Göteson et al.^1^** | | |
| --- | --- | --- | --- | --- | --- | --- |
| **Assay** | **OR** | **95% CI** | **p** | **OR** | **95% CI** | **p** |
| BetaNGF |  |  |  |  |  |  |
| SBP-S | 1.36 | 0.98-1.93 | 0.07 | 0.96 | 0.72-1.30 | 0.80 |
| SBP-G | 0.68 | 0.44-1.04 | 0.08 | 0.79 | 0.52-1.17 | 0.24 |
| CDH3 |  |  |  |  |  |  |
| SBP-S | N/A | N/A | N/A | 0.84 | 0.61-1.13 | 0.25 |
| SBP-G | N/A | N/A | N/A | 1.03 | 0.69-1.49 | 0.89 |
| EZR |  |  |  |  |  |  |
| SBP-S | 0.94 | 0.62-1.47 | 0.79 | 1.12 | 0.78-1.68 | 0.57 |
| SBP-G | 0.11 | 0.75-1.65 | 0.63 | 1.50 | 1.00-2.34 | 0.06 |
| GDNF |  |  |  |  |  |  |
| SBP-S | N/A | N/A | N/A | N/A | N/A | N/A |
| SBP-G | N/A | N/A | N/A | N/A | N/A | N/A |
| IL12 |  |  |  |  |  |  |
| SBP-S | 1.05 | 0.77-1.43 | 0.74 | 0.95 | 0.71-1.27 | 0.73 |
| SBP-G | 0.87 | 0.58-1.29 | 0.48 | 1.08 | 0.74-1.59 | 0.69 |
| NTRK3 |  |  |  |  |  |  |
| SBP-S | 0.90 | 0.65-1.24 | 0.52 | 0.83 | 0.62-1.10 | 0.20 |
| SBP-G | 0.72 | 0.46-1.10 | 0.14 | 0.81 | 0.53-1.22 | 0.31 |

Results from logistic regression models with patient/control as the dependent variable. Covariates: Age, sex, body mass index, CSF/serum albumin ratio, nicotine use. GDNF was excluded in both studies due to a high proportion of individuals with values under the detection limit. CDH3 was excluded in the present study for the same reason. The other four proteins were included in both studies.

*Abbreviations:* BetaNGF, beta-nerve growth factor. CDH3, cadherin-3. EZR, ezrin. CI, confidence interval. GDNF, glial cell line-derived neurotrophic factor. IL12, interleukin-12. N/A, not available (due to >25% of individuals with values below limit of detection in both the patient and the control groups). NTRK3, NT-3 growth factor receptor. OR, odds ratio. SBP-G, St. Göran bipolar project Gothenburg. SBP-S, St. Göran bipolar project Stockholm.

^1^ Cerebrospinal fluid proteomics targeted for central nervous system processes in bipolar disorder. Göteson A, Isgren A, Jonsson L, Sparding T, Smedler E, Pelanis A, Zetterberg H, Jakobsson J, Pålsson E, Holmén-Larsson J, Landén M. *Molecular Psychiatry*. 2021. Dec;26(12):7446-7453.

**Supplementary table 5**. Linear and ordinal regressions for CSF growth hormone and clinical variables.

|  | **SBP-S** | | | | **SBP-G** | | | |
| --- | --- | --- | --- | --- | --- | --- | --- | --- |
| **Clinical feature^1^** | **OR** | **CI** | **SE** | **p** | **OR** | **CI** | **SE** | **p** |
| Manic/hypomanic episodes | 0.88 | 0.55-1.37 | 0.23 | 0.570 | 0.90 | 0.44-1.85 | 0.37 | 0.765 |
| Depressive episodes | 0.59 | 0.37-0.93 | 0.24 | **0.026** | 1.21 | 0.57-2.56 | 0.38 | 0.622 |
| CGI | 0.89 | 0.55-1.44 | 0.25 | 0.645 | 1.48 | 0.69-3.27 | 0.39 | 0.318 |
| GAF-s | 1.12 | 0.72-1.76 | 0.23 | 0.614 | 0.57 | 0.26-1.18 | 0.38 | 0.133 |
| GAF-f | 0.96 | 0.61-1.52 | 0.23 | 0.859 | 0.60 | 0.27-1.28 | 0.39 | 0.188 |
| **Drugs^2^** | **β** | **t** | **SE** | **p** | **β** | **t** | **SE** | **p** |
| Lithium use | -0.11 | -0.69 | 0.16 | 0.489 | -0.23 | -0.93 | 0.24 | 0.356 |
| Antipsychotics | -0.20 | -1.03 | 0.20 | 0.304 | -0.84 | -3.78 | 0.22 | **<0.001** |
| Anticonvulsants | -0.11 | -0.67 | 0.17 | 0.507 | 0.03 | 0.11 | 0.23 | 0.910 |
| Antidepressants | -0.28 | -1.78 | 0.16 | 0.077 | -0.08 | -0.37 | 0.23 | 0.710 |

^1^ Ordinal regression models with categorized clinical feature as dependent variable. Covariates: Age, sex, BMI, CSF/serum albumin ratio. ^2^Linear regression models with growth hormone as dependent variable. Covariates: Age, sex, CSF/serum albumin ratio.

*Abbreviations*: BMI, body mass index. CGI, Clinical Global Impression rating scale. CI, confidence interval for OR. CSF, cerebrospinal fluid. GAF, Global Assessment of Functioning scale divided into functional level (GAF-f) and symptom severity (GAF-s). OR, odds ratio. SBP-G, St. Göran bipolar project Gothenburg. SBP-S, St. Göran bipolar project Stockholm. SE, standard error.

**Supplementary table 6**. Logistic regressions showing the association between growth hormone and bipolar disorder, when patients with antipsychotic treatment were excluded.

| **SBP-S (n=179)** | **β** | **SE** | **OR** | **95% CI** | **p** |
| --- | --- | --- | --- | --- | --- |
| GH | -0.57 | 0.24 | 0.57 | 0.35-0.90 | **0.019** |
| Age | -0.01 | 0.01 | 0.99 | 0.96-1.01 | 0.400 |
| Male sex | -0.98 | 0.38 | 0.37 | 0.17-0.78 | **0.010** |
| BMI | 0.01 | 0.05 | 1.01 | 0.91-1.12 | 0.913 |
| Nicotine use | 1.01 | 0.34 | 2.75 | 1.42-5.45 | **0.003** |
| **CSF/serum albumin ratio** | 0.08 | 0.09 | 1.08 | 0.91-1.30 | 0.373 |
| **SBP-G (n=101)** | **β** | **SE** | **OR** | **95% CI** | **p** |
| GH | -0.43 | 0.34 | 0.65 | 0.32-1.25 | 0.209 |
| Age | -0.03 | 0.02 | 0.97 | 0.93-1.01 | 0.099 |
| Male sex | -0.10 | 0.50 | 0.37 | 0.13-0.98 | 0.051 |
| BMI | -0.04 | 0.07 | 0.96 | 0.83-1.11 | 0.577 |
| Nicotine use | 0.77 | 0.46 | 2.15 | 0.88-5.48 | 0.099 |
| **CSF/serum albumin ratio** | 0.10 | 0.12 | 1.10 | 0.86-1.43 | 0.445 |

Case–control status is dependent variable. Age, sex, BMI, CSF/serum albumin ratio and nicotine use are covariates.

*Abbreviations*: BMI, body mass index. CI, confidence interval. CSF, cerebrospinal fluid. GH, growth hormone. OR, odds ratio. SBP-G, St. Göran bipolar project Gothenburg. SBP-S, St. Göran bipolar project Stockholm. SE, standard error.

**Supplementary table 7**. Logistic regressions showing the association between growth hormone and bipolar disorder, when patients with MADRS score of 13 or higher, and/or YMRS score of 14 or higher were excluded.

| **SBP-S (n=197)** | **β** | **SE** | **OR** | **95% CI** | **p** |
| --- | --- | --- | --- | --- | --- |
| GH | -0.73 | 0.25 | 0.48 | 0.29-0.78 | **0.003** |
| Age | -0.01 | 0.01 | 0.99 | 0.97-1.02 | 0.586 |
| Male sex | -0.96 | 0.37 | 0.38 | 0.18-0.77 | **0.009** |
| BMI | -0.02 | 0.05 | 0.98 | 0.89-1.10 | 0.773 |
| Nicotine use | 0.94 | 0.34 | 2.57 | 1.33-5.10 | **0.006** |
| **CSF/serum albumin ratio** | 0.15 | 0.09 | 1.16 | 0.99-1.39 | 0.075 |
| **SBP-G (n=116)** | **β** | **SE** | **OR** | **95% CI** | **p** |
| GH | -0.86 | 0.34 | 0.42 | 0.21-0.81 | **0.012** |
| Age | -0.06 | 0.02 | 0.94 | 0.90-0.98 | **0.002** |
| Male sex | -1.35 | 0.54 | 0.26 | 0.08-0.72 | **0.013** |
| BMI | 0.02 | 0.07 | 1.02 | 0.89-1.17 | 0.826 |
| Nicotine use | 0.70 | 0.46 | 2.00 | 0.83-5.01 | 0.127 |
| **CSF/serum albumin ratio** | 0.12 | 0.13 | 1.13 | 0.89-1.46 | 0.332 |

Case–control status is dependent variable. Age, sex, BMI, CSF/serum albumin ratio and nicotine use are covariates.

*Abbreviations*: BMI, body mass index. CI, confidence interval. CSF, cerebrospinal fluid. GH, growth hormone. MADRS, Montgomery-Åsberg Depression Rating Scale. OR, odds ratio. SBP-G, St. Göran bipolar project Gothenburg. SBP-S, St. Göran bipolar project Stockholm. SE, standard error. YMRS, Young Mania Rating Scale.

**Supplementary table 10**. Summary of methods and results of the present study and results from two previous studies measuring CSF concentrations of IL-8^I^, MCP-1^II^, and YKL-40^II^ in bipolar disorder patients and controls.

|  | **SBP-S**  **ELISA**  *(study I-II, see below)* | **SBP-S**  **PEA**  *(present study)* | **SBP-G**  **PEA**  *(present study)* |
| --- | --- | --- | --- |
| IL-8 | **BD>CTRL**^1^  (p=0.042) | BD>CTRL^3^  (p=0.08) | BD<CTRL^3^  (p=0.66) |
| MCP-1 | **BD>CTRL**^2^  (p=0.004) | BD>CTRL^3^  (p=0.07) | BD>CTRL^3^  (p=0.82) |
| YKL-40 | **BD>CTRL**^2^  (p=0.014) | **BD>CTRL**^3^  (p=0.03) | BD<CTRL^3^  (p=0.54) |
| ^1^Analysis of covariance (ANCOVA) with age and CSF/serum albumin ratio as covariates. ^2^ Linear regression with CSF protein concentration as dependent variable and age, sex, body mass index, nicotine use and CSF/serum albumin ratio and C-reactive protein as covariates. ^3^ Logistic regression with BD/CTRL as dependent variable and age, sex, body mass index, nicotine use and CSF/serum albumin ratio as covariates.  > shows that CSF concentrations for patients were higher compared with controls. < shows that CSF concentrations for patients were lower compared with controls. Results in bold are significant at a p<0.05 level.  *Abbreviations*: BD, bipolar disorder. CTRL, controls. ELISA, enzyme-linked immunosorbent assays (electrochemiluminescence or colorimetric). PEA, proximity extension assay. SBP-G, St. Göran bipolar project Gothenburg. SBP-S, St. Göran bipolar project Stockholm. | | | |

I. Isgren A, Jakobsson J, Palsson E, Ekman CJ, Johansson AG, Sellgren C *et al.* Increased cerebrospinal fluid interleukin-8 in bipolar disorder patients associated with lithium and antipsychotic treatment. *Brain Behav Immun* 2015; **43:** 198-204

II. Jakobsson J, Bjerke M, Sahebi S, Isgren A, Ekman CJ, Sellgren C *et al.* Monocyte and microglial activation in patients with mood-stabilized bipolar disorder. *J Psychiatry Neurosci* 2015; **40**(4)**:** 250-258.

**
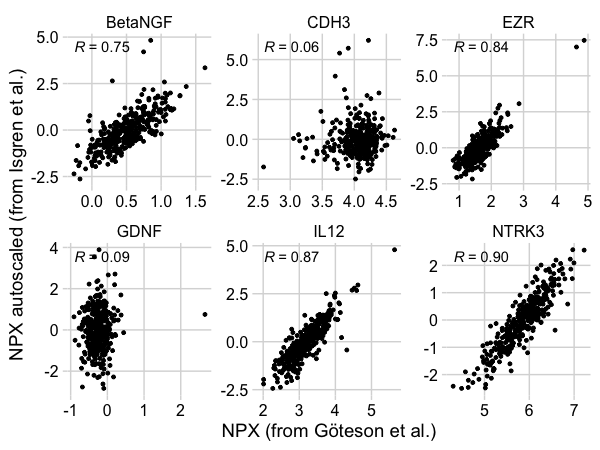
**

**Supplementary figure 1**. Correlations between protein concentrations in the present study and in a previously published study (Göteson et al.^1^) for the six overlapping assays. GDNF was excluded in both studies due to a high proportion of individuals with values under the detection limit. CDH3 was excluded in the present study for the same reason. The other four proteins were included in both studies. In the present study (Isgren et al.), NPX values were centered at zero and scaled to unit variance (standard deviation = 1, i.e., autoscaling). No zero centering or scaling was done in the study by Göteson et al.

*Abbreviations:* BetaNGF, beta-nerve growth factor. CDH3, cadherin-3. EZR, ezrin. GDNF, glial cell line-derived neurotrophic factor. IL12, interleukin-12. NPX, normalized protein expression. NTRK3, NT-3 growth factor receptor. R, Pearson correlation coefficient.

^1^ Cerebrospinal fluid proteomics targeted for central nervous system processes in bipolar disorder. Göteson A, Isgren A, Jonsson L, Sparding T, Smedler E, Pelanis A, Zetterberg H, Jakobsson J, Pålsson E, Holmén-Larsson J, Landén M. *Molecular Psychiatry*. 2021. Dec;26(12):7446-7453.

**
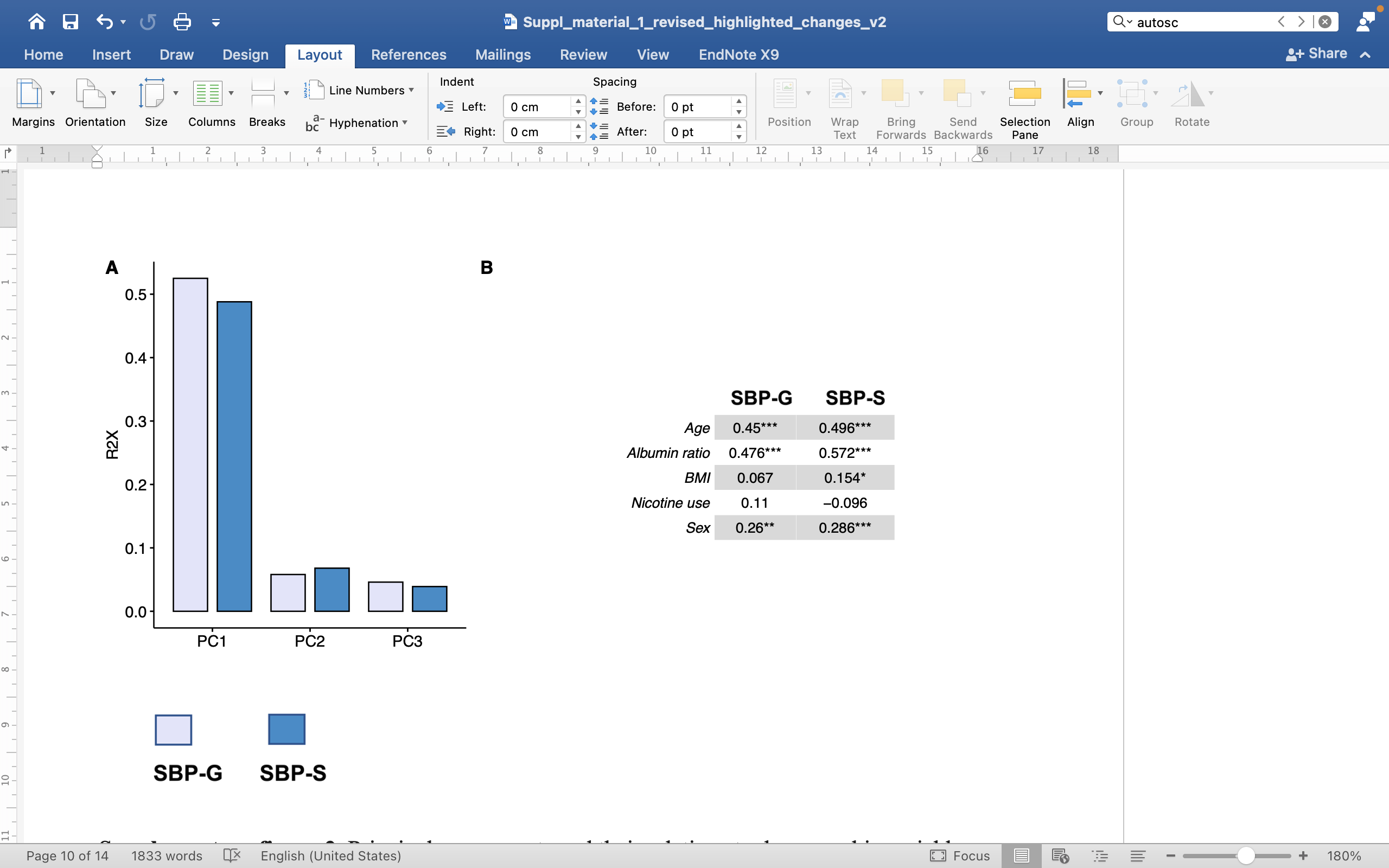
**

**Supplementary figure 2**. Principal components and their relations to demographic variables.

**A**. The explained variation for the three first principal components from a principal component analysis in SBP-S and SBP-G. The y-axis (R2X) shows the fraction of the total variation of the protein concentrations in the dataset that can be explained by each component. **B**. Spearman correlation coefficients for first principal components and demographic variables. Legend: Significance levels: *<0.05, **<0.01, ***<0.001.

*Abbreviations*: BMI, body mass index. PC, principal component. SBP-G, St. Göran bipolar project Gothenburg. SBP-S, St. Göran bipolar project Stockholm.

**SBP-S**

**SBP-G**

**Supplementary figure 3**. Principal component analyses (PCA) of cerebrospinal fluid protein concentrations in bipolar disorder patients and controls.

*Abbreviations*: BD, bipolar disorder. CTRL, control. SBP-G, St. Göran bipolar project Gothenburg. SBP-S, St. Göran bipolar project Stockholm.


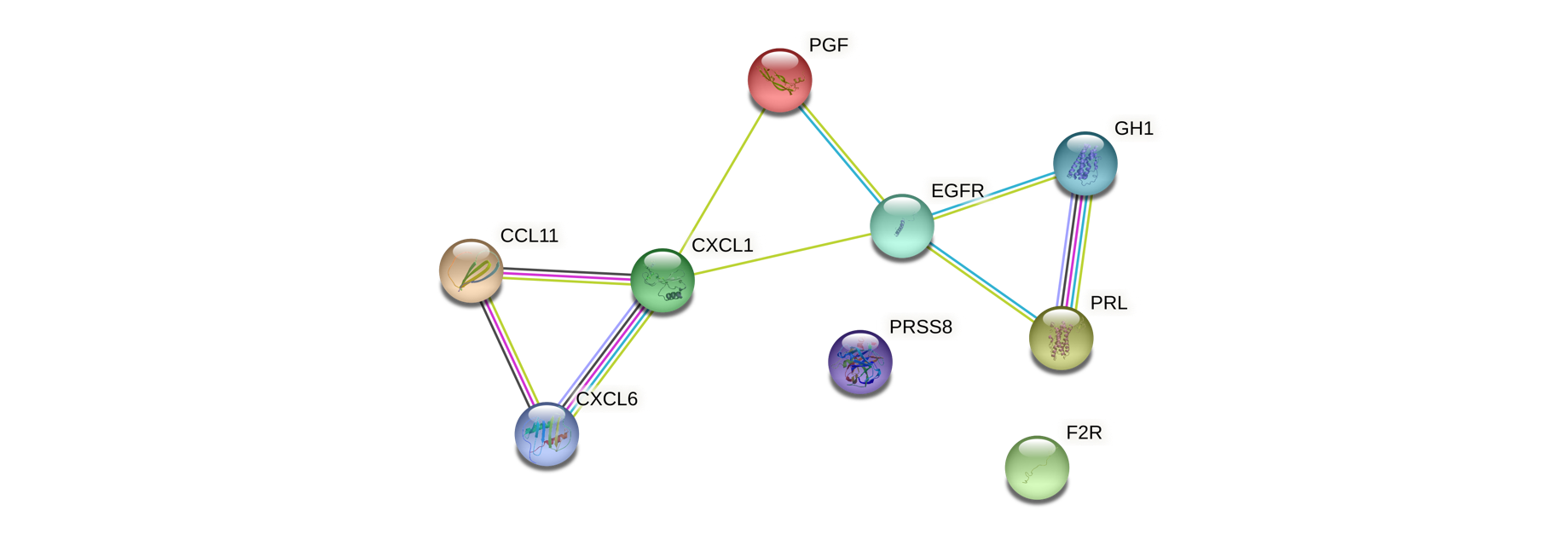


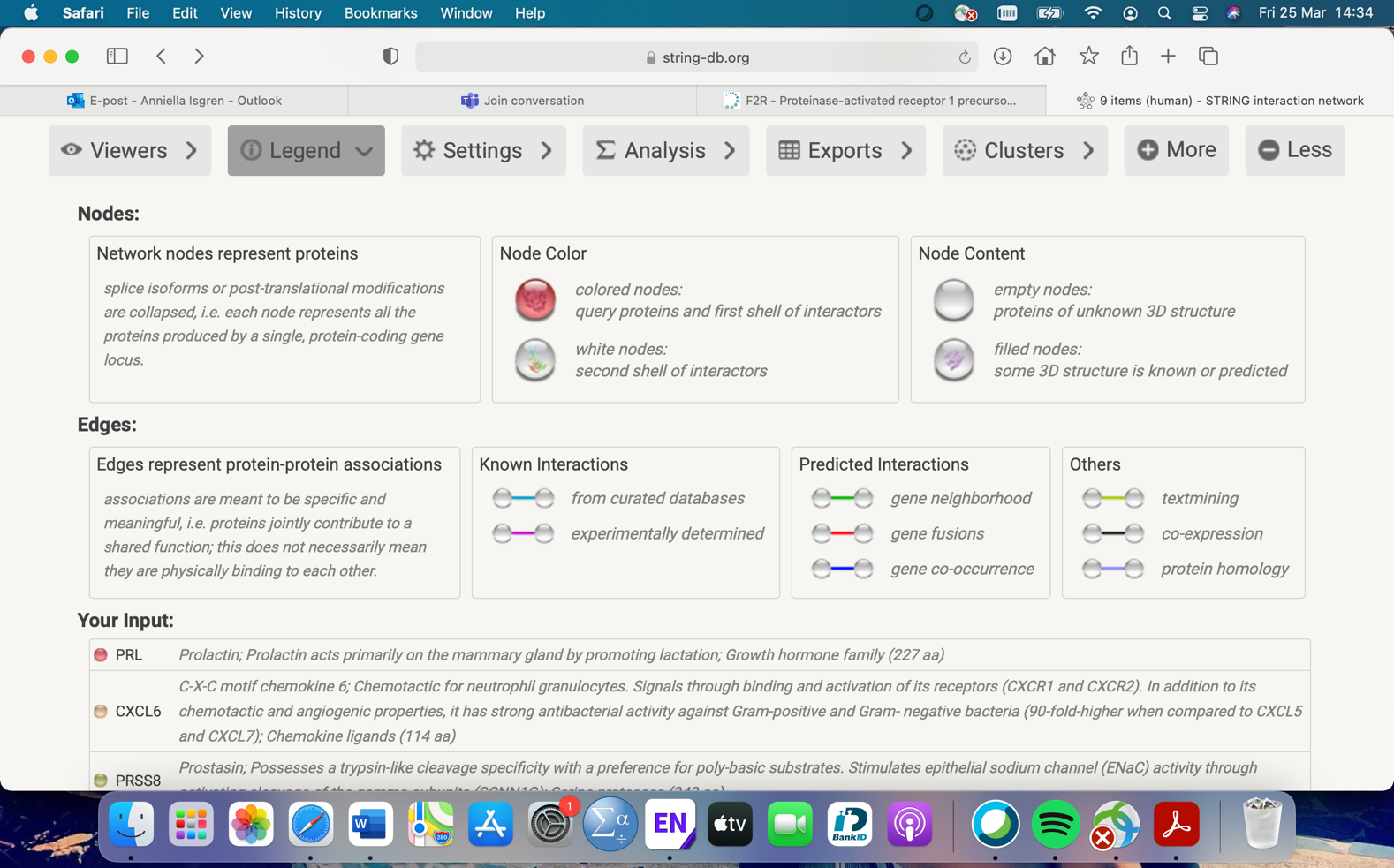


**Supplementary figure 4**. Graph of protein–protein interactions for growth hormone and the eight proteins for which there was a significant difference in CSF concentrations between patients and controls in one cohort, and an estimate in the same direction (however not statistically significant) in the other cohort. Results from STRING database of known and predicted protein–protein interactions.

*Abbreviations*: CCL11, eotaxin-1. PGF, placenta growth factor. CXCL1, C-X-C motif chemokine 1. CXCL6, C-X-C motif chemokine 6. EGFR, epidermal growth factor receptor. F2R, proteinase-activated receptor 1. GH1, growth hormone. PRL, prolactin. PRSS8, prostasin.


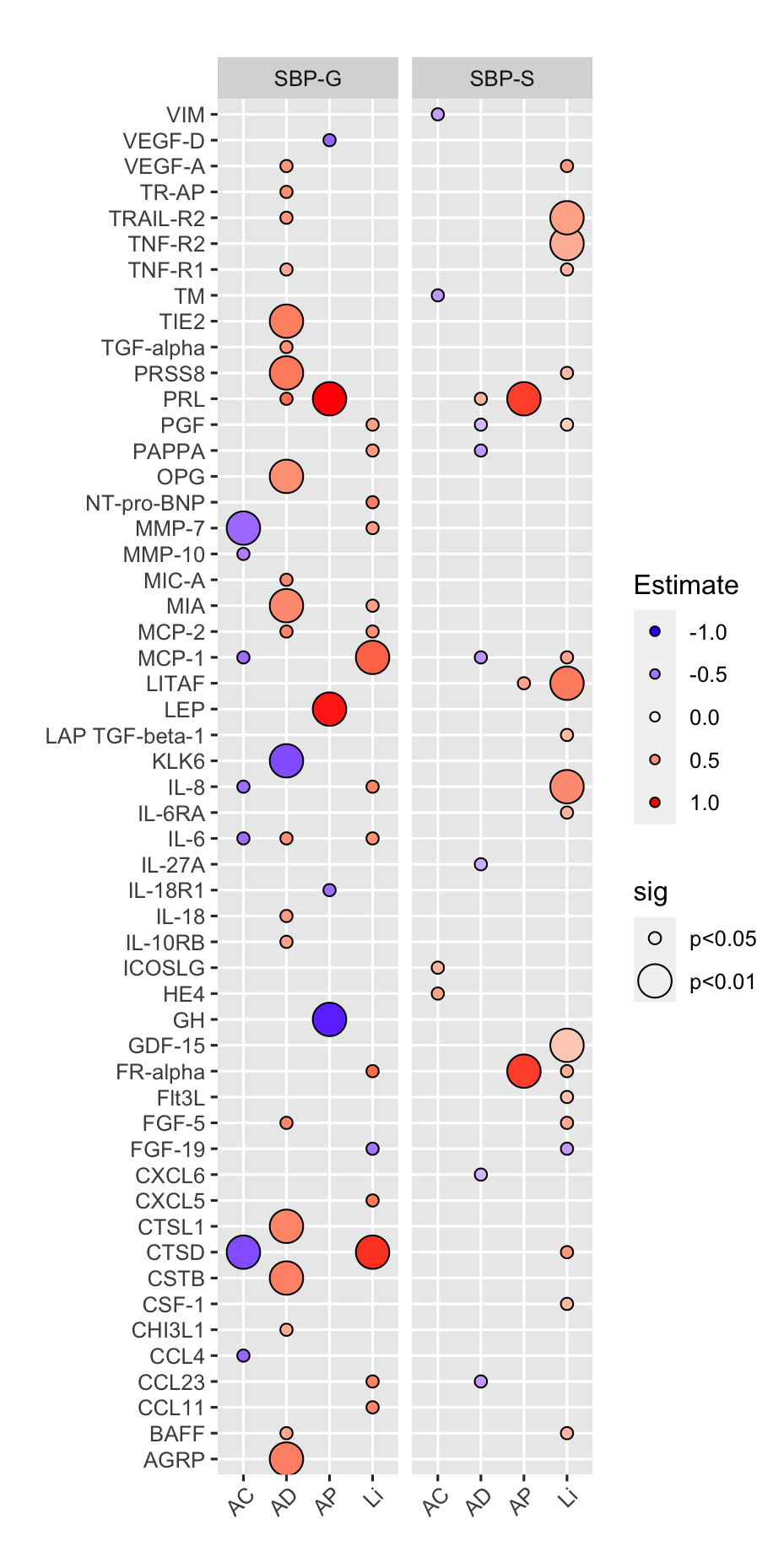


**Supplementary figure 5**. Heatmap visualizing the associations between cerebrospinal fluid protein concentrations and psychiatric drug groups. Proteins with a p<0.05 in one or two cohorts are included in the figure. Statistics from linear regression models with protein concentrations as dependent variables and age, sex, and CSF/serum albumin ratio as covariates.

*Abbreviations*: AC, anticonvulsants. AP, antipsychotics. AD, antidepressants. Li, lithium. SBP-G, St. Göran bipolar project Gothenburg. SBP-S, St. Göran bipolar project Stockholm.

**Supplementary figure 6**. Histogram of between-fluid (CSF–serum) protein correlations for the 105 proteins analyzed in CSF in the present study and in serum in a previous study^1^.

Count shows the number of proteins in each size range of Spearman’s correlation coefficient.

*Abbreviations*: SBP, St. Göran bipolar project.

^1^ Göteson A, Isgren A, Sparding T, Holmén-Larsson J, Jakobsson J, Pålsson E, Landén M. A serum proteomic study of two case-control cohorts identifies novel biomarkers for bipolar disorder. *Translational Psychiatry* 2022 Feb 8;12(1):55)
